# An Effective Method for Calculation of Mutual Inductance Between Rectangular Coils at Arbitrary Positions in Space

**DOI:** 10.3390/s25113265

**Published:** 2025-05-22

**Authors:** Junlin Chen, Guofeng Yao, Min Wang, Liming Zhou, Kuiyang Gao, Peilei Zhou, Ruiyao Liu

**Affiliations:** 1 Key Laboratory of CNC Equipment Reliability, Ministry of Education, School of Mechanical and Aerospace Engineering, Jilin University, Changchun 130025, China; cjl19990919@163.com (J.C.); lmzhou@jlu.edu.cn (L.Z.); gaoky21@mails.jlu.edu.cn (K.G.); ruiyaoliu@126.com (R.L.); 2 School of Mechanical and Aerospace Engineering, Jilin University, Changchun 130025, China; 3 College of Transportation, Jilin University, Changchun 130025, China; zhoupeilei11@jlu.edu.cn

**Keywords:** mutual inductance, arbitrary positions, rectangular coil, Neumann formula, magnetic field distribution

## Abstract

Electromagnetic torques generated by mutual inductance between energized coils are widely used in aerospace applications, especially for solar panel deployment. Accurate and rapid acquisition of mutual inductance between coils is essential to provide the necessary electromagnetic force. Therefore, based on the Kalantarov–Zeitlin method and the Neumann formula, this paper presents a straightforward and efficient calculation method for mutual inductance between rectangular coils positioned arbitrarily in space. Building on this foundation, we develop a calculation method for mutual inductance between rectangular multi-turn coils using the principle of superposition. The accuracy of the proposed method’s calculations is validated using data from the published literature, and the computation time is compared with that of other methods. To further validate the accuracy of the computational method proposed in this paper, a rectangular multi-turn coil mutual inductance measurement platform has been constructed. The results indicate that the computation time of the proposed method is shorter, and the calculation outcomes closely align with those obtained from other methods as well as experimental measurements. Furthermore, the calculation accuracy exceeds 95%, providing a reliable basis for determining the electromagnetic force required for the deployment of the solar array driven by electromagnetism.

## 1. Introduction

**Figure 1:**
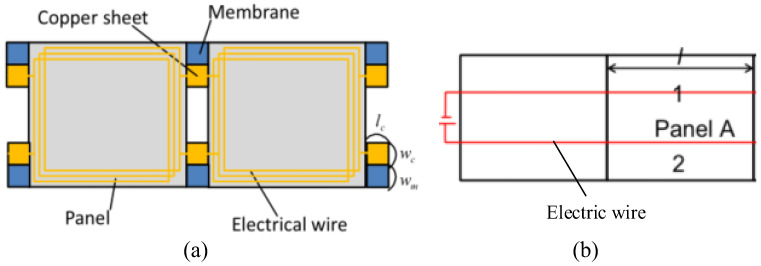
Electromagnetically driven solar panel deployment schematic: (**a**) Coil generates electromagnetic force, (**b**) Electromagnetic force generated by electric wires.

With the rapid advancement in aviation technology, the types and applications of spacecraft are becoming increasingly diverse. To continuously, safely, and reliably provide power for spacecraft operations in space, solar energy has emerged as the optimal choice [1]. Solar panels, which serve as the primary devices for collecting solar energy, have become essential components of spacecraft design [2,3,4]. Traditional drive technologies primarily utilize motors, springs, and pneumatic mechanisms to facilitate the deployment of solar panels [5,6,7]. However, these methods present challenges, including complex structures and potential impacts on spacecraft performance. To ensure the normal operation and service of the deployment mechanism, electromagnetic drive control systems [8,9,10] have emerged as a prominent area of research in recent years. Compared to traditional driving methods, this approach is more straightforward, requiring only the installation of energized coils on the surface of solar panels. By utilizing the principle of electromagnetic induction, this method facilitates the contactless unfolding of solar panels through the electromagnetic force generated between the coils, as illustrated in Figure 1. The electromagnetic force in this technique is a non-contact field force, providing advantages such as continuity, reversibility, synchronization, and controllability [11]. The key to generating the electromagnetic force lies in accurately determining the mutual inductance between the coils installed on the surface of the solar panel.

The finite element method (FEM) and theoretical calculations are the primary techniques for determining the mutual inductance between coils. FEM is capable of modeling and analyzing coils with various structures and accurately simulating mutual inductance in different environments, making it an essential approach for obtaining this parameter. In contrast, theoretical calculations enable the determination of mutual inductance by substituting model parameters into a calculation formula. Consequently, using theoretical methods to calculate mutual inductance can streamline the process, reduce computational workload, and enhance efficiency. This approach also presents opportunities for further optimization. Currently, numerous studies on methods for calculating mutual inductance exist, primarily based on the Biot–Savart law [12], elliptic integrals [13,14], Bessel functions [15], Fourier transforms [16], and the Neumann formula [17].

The calculation of mutual inductance between circular coils is a well-established and widely utilized concept. Maxwell [18] proposed a classical method for determining the mutual inductance between parallel and coaxial circular coils, which serves as a significant reference for subsequent studies on this topic. Babic et al. [19] derived a new formula for calculating the mutual inductance between arbitrarily positioned inclined circular coils, employing the vector potential method and comparing it with the classical formula presented by Grover [20]. The results indicate that the new formula is applicable for mutual inductance calculations across various fields. K.V. Poletkin et al. [21] utilized the Kalantarov–Zeitlin method to compute the mutual inductance between circular coils through line integration, providing an alternative to the expressions proposed by Grover and Babic. S. Gao et al. [22] applied the Neumann formula to model the mutual inductance calculation for circular multi-turn coils and introduced a multi-to-one wireless power transmission (WPT) spherical actuator attitude measurement method. Experimental results demonstrate that the angle error derived from the proposed calculation model is effectively maintained within 2%. X. Qiao et al. [23] established a mutual inductance calculation model for circular spiral tube coils using the Neumann formula and analyzed the impact of transmission distance on the transmission characteristics of the rotary ultrasonic machining (RUM) system.

However, compared to circular coils, rectangular coils exhibit a lower fluctuation rate of mutual inductance during positional changes [24,25]. Additionally, the flat shape of solar panels on the spacecraft makes rectangular coils more suitable for mounting on their surfaces. Consequently, rectangular coils are more effective for harnessing electromagnetic force in the deployment technology of electromagnetically driven solar panels. It is essential to systematically investigate the calculation methods for mutual inductance between rectangular coils to ensure the reliable acquisition of electromagnetic force. Sugawara et al. [9] proposed a method for deploying solar panels on low Earth orbit (LEO) satellites by installing rectangular coils on the surface of the panels and utilizing the mutual inductance between the geomagnetic field and the coils to generate electromagnetic force. Y. Ji et al. [26] introduced a mutual inductance calculation formula for rectangular coils in arbitrary orientations, based on the Neumann formula and rotation matrices. Results indicate that this method has a lower relative error and significantly improved calculation speed compared to other approaches. F. Durmus et al. [27] proposed a calculation method for the mutual inductance of square and hexagonal coils based on the Neumann integral, which involves calculating the mutual inductance of two arbitrarily arranged straight wires. H. Tavakkoli et al. [28] developed a formula for calculating the mutual inductance of coaxial hexagonal and octagonal planar spiral coils using the Biot–Savart law and the magnetic flux density method. They validated the feasibility of the derived formula through experiments and simulations. M. Song et al. [29] utilized the Neumann formula to propose a calculation method for the self and mutual inductance of multi-turn zero-flux rectangular coils. This method is used to derive the analytical formula for the electromagnetic force of the electrodynamic suspension system.

The calculation of mutual inductance between rectangular coils primarily relies on the magnetic flux density method [30] and the Neumann formula. Both methods suffer from the inability to quickly and accurately localize the position and attitude of the coil in space. If the spatial position of the coils involves rotation or more complex changes, this requirement significantly increases computation time and reduces overall efficiency. When solar panels are deployed, it is essential to quickly and accurately adjust their positions according to the spacecraft’s instructions. Therefore, a calculation method that can swiftly and precisely determine the mutual inductance between rectangular coils at arbitrary positions in space is needed to ensure the effective acquisition of electromagnetic force.

The Kalantarov–Zeitlin method demonstrates that the calculation of mutual inductance between a circular primary filament and any secondary filament—regardless of its shape or position relative to the primary filament—can be simplified to a line integral [21]. This approach significantly reduces the computation time required for calculations in mutual inductance theory. In this paper, we utilize this method with the Neumann formula and a transformation to rectangular coordinates to propose a simple and efficient method for calculating the mutual inductance between rectangular coils positioned at arbitrary locations in space. Our study derives the formula for calculating mutual inductance specifically for rectangular spiral coils by applying the principle of superposition, which is particularly relevant for applications in electromagnetic drives. The computational accuracy and speed of the proposed method are validated by comparing the results with the Neumann formula and the magnetic flux density method. Additionally, the mutual inductance between two rectangular helical coils with varying positional configurations is measured using an impedance analyzer and a custom-built acrylic moving platform. This process is simulated using an ANSYS electronic finite element script. By analyzing the changes in coil position in different positions and attitudes, the theoretical calculated values of mutual inductance of the method proposed in this paper are highly consistent with the experimental and simulation results, which indicates the accuracy of the calculation method proposed in this paper. Table 1 presents the symbols used in this paper along with their definitions.

The specific work of this paper is structured as follows: Section 2 derives the expression of mutual inductance calculation between single-turn rectangular coils at arbitrary spatial positions through Neumann’s formula and the Kalantarov–Zeitlin method, on the basis of which the mutual inductance calculation model of multi-turn rectangular coils is obtained from the superposition principle. Section 3 compares the mutual inductance data in the published literature with the method proposed in this paper to verify the accuracy and simplicity of the calculation method in this paper. Section 4 uses a self-made mobile platform with an impedance analyzer to conduct experimental tests of mutual inductance and numerical simulation to further verify the accuracy of the calculation method in this paper. Section 5 discusses and analyzes the computational results obtained from experiments, simulations, and calculations.

## 2. Mutual Inductance Calculation of Rectangular Coil

The electromagnetic force in electromagnetic drive technology fundamentally represents the interaction between two coils. The expression for this force is derived using the principle of virtual displacement [31]:

(1) $$\left\{\begin{array}{l} F_{x}=\frac{\partial M}{\partial x}I_{1}I_{2}\\ F_{y}=\frac{\partial M}{\partial y}I_{1}I_{2}\\ F_{z}=\frac{\partial M}{\partial z}I_{1}I_{2} \end{array}\right.$$

where *I*_1_ and *I*_2_ represent the currents in the two coils. From Equation (1), it can be observed that the electromagnetic force in the three directions consists of two components, i.e., the current and the mutual inductance gradient of the coils. A critical aspect of determining the electromagnetic force is accurately calculating the mutual inductance between the rectangular coils.

**Figure 2:**
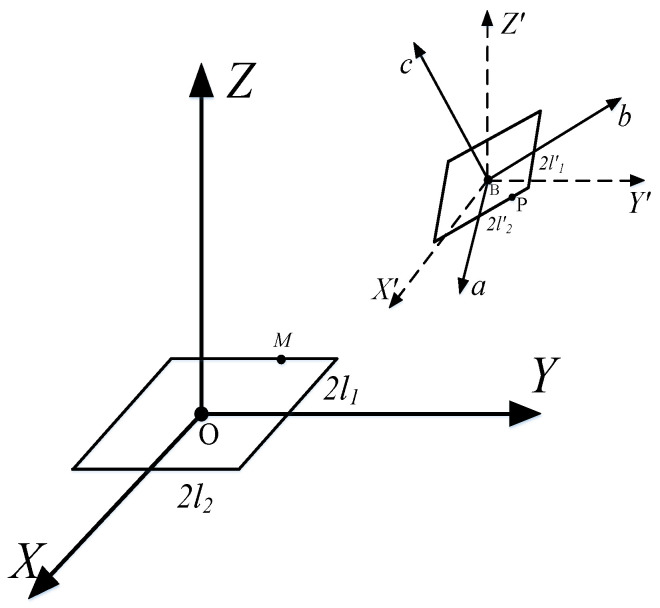
General scheme of arbitrarily positioning two rectangular coils with respect to each other: M and P represent arbitrary points on the primary coil and secondary coil, respectively.

This paper primarily investigates the mutual inductance between arbitrarily positioned rectangular coils, as illustrated in Figure 2. The side lengths of the primary coil and secondary coil are denoted as 2*l*_1_, 2*l*_2_ and 2*l’*_1_, 2l*’*_2_, respectively. A Cartesian coordinate system (*O-XYZ*) is established with the center O of the primary coil as the origin, and the rectangular plane is situated on the XOY plane. Similarly, a body-fixed coordinate system (*B-X’Y’Z’*) is established with the center B of the secondary coil.

### 2.1. Coordinate Transformation

**Figure 3:**
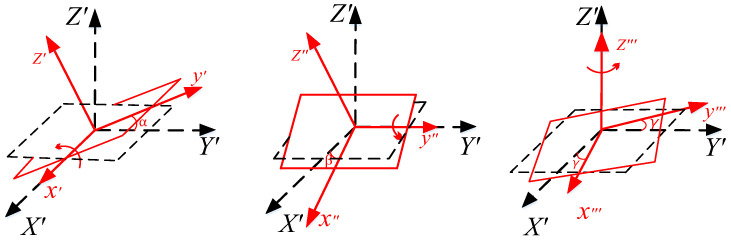
Rectangular coils at arbitrary positions and attitudes. Red indicates the change in the position of the rectangle.

As illustrated in Figure 3, the rotation angle is defined by the Euler angles around the *X’-*, *Y’-*, and *Z’*-axes, which are, respectively, denoted as *α*, *β*, and *γ*. The total rotation is divided into three steps: rotation around the *X’*-axis by *α*, rotation around the *Y’*-axis by *β*, and rotation around the *Z’*-axis by *γ*.

The rotation matrix represented by the Euler angles is obtained as follows:

(2) $$R_{\alpha \beta \gamma }=R_{z}\left(\gamma \right)R_{y}\left(\beta \right)R_{x}\left(\alpha \right)=\left(\begin{array}{ccc} R_{11} & R_{12} & R_{13}\\ R_{21} & R_{22} & R_{23}\\ R_{31} & R_{32} & R_{33} \end{array}\right)$$

The rotation matrix R is expressed as follows:

(3) $$R_{x}\left(\alpha \right)=\left(\begin{array}{ccc} 1 & 0 & 0\\ 0 & \cos\alpha & -\sin\alpha \\ 0 & \sin\alpha & \cos\alpha \end{array}\right)$$

(4) $$R_{y}\left(\beta \right)=\left(\begin{array}{ccc} \cos\beta & 0 & \sin\beta \\ 0 & 1 & 0\\ -\sin\beta & 0 & \cos\beta \end{array}\right)$$

(5) $$R_{z}\left(\gamma \right)=\left(\begin{array}{ccc} \cos\theta & -\sin\theta & 0\\ \sin\theta & \cos\theta & 0\\ 0 & 0 & 1 \end{array}\right)$$

The coordinates of arbitrary points on the secondary coil under the *B-X’Y’Z’* coordinate system are as follows:

(6) $$\left(\begin{array}{l} x^{\prime }\\ y^{\prime }\\ z^{\prime } \end{array}\right)=\left(\begin{array}{ccc} R_{11} & R_{12} & R_{13}\\ R_{21} & R_{22} & R_{23}\\ R_{31} & R_{32} & R_{33} \end{array}\right)\left(\begin{array}{l} x^{\prime \prime }\\ y^{\prime \prime }\\ z^{\prime \prime } \end{array}\right)$$

Finally, according to Equations (2)–(6), the coordinates of arbitrary points on the secondary coil under the *O-XYZ* coordinate system are expressed as follows:

(7) $$\left(\begin{array}{l} x\\ y\\ z \end{array}\right)=\left(\begin{array}{l} x^{\prime }\\ y^{\prime }\\ z^{\prime } \end{array}\right)+\left(\begin{array}{l} x_{B}\\ y_{B}\\ z_{B} \end{array}\right)=\left(\begin{array}{ccc} R_{11} & R_{12} & R_{13}\\ R_{21} & R_{22} & R_{23}\\ R_{31} & R_{32} & R_{33} \end{array}\right)\left(\begin{array}{l} x^{\prime \prime }\\ y^{\prime \prime }\\ z^{\prime \prime } \end{array}\right)+\left(\begin{array}{l} x_{B}\\ y_{B}\\ z_{B} \end{array}\right)$$

Equation (6) is the mathematical model of the nonlinear three-dimensional coordinate transformation.

### 2.2. Neumann Formula

The Neumann formula is a fundamental method for calculating mutual inductance. It simplifies the computation of mutual inductance between two coils of arbitrary positions and shapes into a line integral form.

As shown in Figure 1, when there is current *I* in the primary coil l, the relationship between the vector magnetic potential A generated by the primary coil l on the secondary coil l’ and the magnetic induction intensity B is expressed as follows:

(8) B=∇×A

According to the Biot–Savart law, the magnetic induction intensity B generated by the coil is expressed as follows:

(9) $$B=\frac{\mu_{0}}{4\pi }\int_{l}\frac{Idl\times R}{R^{3}}$$

(10) $$\nabla \left(\frac{1}{R}\right)=-\frac{1}{R^{3}}R$$

(11) $$B=-\frac{\mu_{0}}{4\pi }\int_{l}Idl\times \nabla (\frac{1}{R})$$

where μ_0_ = 4π × 10^−7^ H/m is the magnetic conductivity in vacuum and R represents the distance between the arbitrary point on the primary coil l and the arbitrary point on the secondary coil l’.

According to the vector algorithm,

(12) $$\nabla \times \left(\frac{Idl}{R}\right)=\nabla \left(\frac{1}{R}\right)\times Idl+\frac{1}{R}\nabla \times Idl$$

(13) $$\frac{1}{R}\nabla \times Idl=0$$

By substituting Equations (12) and (13) into Equation (9), we can obtain the following expression:

(14) $$B=\frac{\mu_{0}}{4\pi }\int_{l}\nabla \times (\frac{Idl}{R})=\nabla \times \frac{\mu_{0}}{4\pi }\int_{l}\left(\frac{Idl}{R}\right)$$

The expression of vector magnetic potential is obtained as follows:

(15) $$A=\frac{\mu_{0}}{4\pi }\int_{l}\left(\frac{Idl}{R}\right)$$

Magnetic linkage *ψ* generated by primary coil l with current I on secondary coil l’ is expressed as follows:

(16) $$\psi =\oint_{l^{\prime }}A\times dl^{\prime }=\frac{\mu_{0}}{4\pi }\oint_{l}\oint_{l^{\prime }}\frac{Idl\cdot dl^{\prime }}{R}$$

By definition, the final mutual inductance between two closed coils of arbitrary shape is expressed as follows:

(17) $$M=\frac{\psi }{I}=\frac{\mu_{0}}{4\pi }\oint_{l}\oint_{l^{\prime }}\frac{dl\cdot dl^{\prime }}{R}$$

### 2.3. Mutual Inductance Calculation

**Figure 4:**
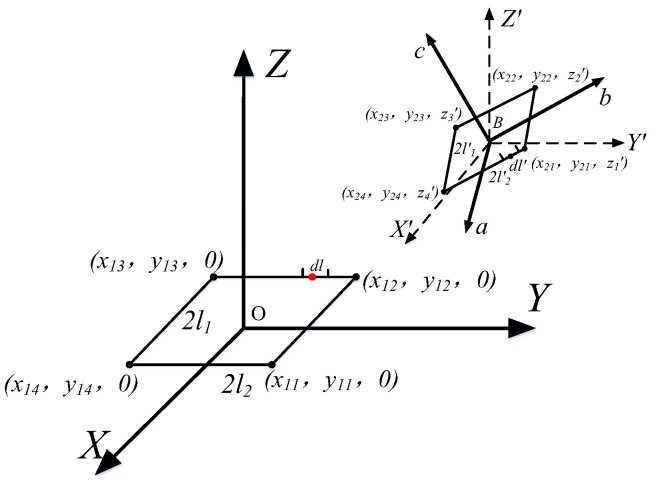
Schematic diagram of coordinates of rectangular coil apex in spatial coordinate system.

As shown in Figure 4, the primary and secondary coils are equated to four segments of wires 2l_1_, 2l_2_, 2l_3_, 2l_4_ and 2l’_1_, 2l’_2_, 2l’_3_, and 2l’_4_, respectively. According to the Neumann formula, the mutual inductance between the two coils can be equated to the mutual inductance between the wires, which reduces the influence of the corner part of the coil on the overall mutual inductance. When two rectangular coils are taken as the line elements *d***l***_a_* and *d***l’***_b_*, the mutual inductance between the two line elements *dM_ab_* is expressed as follows:

(18) $$dM_{ab}=\frac{\mu_{0}}{4\pi }\frac{dl_{a}\cdot d{l^{\prime }}_{b}}{R}$$

The mutual inductance between two rectangular coils is expressed as follows:

(19) $$M_{ab}=\sum_{a=1}^{4}\sum_{b=1}^{4}\frac{\mu_{0}}{4\pi }\frac{dl_{a}\cdot d{l^{\prime }}_{b}}{R}$$

To determine the position of the coil in space, the coordinates of an arbitrary point on the primary coil are (x, y, 0), and the coordinates of an arbitrary point on the secondary coil are (x’, y’, z’). *d***l***_a_·d***l**’*_b_* is calculated as follows:

(20) $$dl_{a}\cdot d{l^{\prime }}_{b}=dxdx^{\prime }+dydy^{\prime }$$

The mutual inductance between two rectangular coils at arbitrary position is calculated by Equations (17)–(20).

(21) $$\begin{array}{ll} M & =\frac{\mu_{0}}{4\pi }\oint_{l}\oint_{l^{\prime }}\frac{dxdx^{\prime }+dydy^{\prime }}{R}\\ & =\frac{\mu_{0}}{4\pi }\left(\int_{x_{1i}}^{x_{1j}}dx\int_{x_{2i}}^{x_{2j}}\frac{dx^{\prime }}{R}+\int_{y_{1i}}^{y_{1j}}dy\int_{y_{2i}}^{y_{2j}}\frac{dy^{\prime }}{R}\right) \end{array}$$

where (*x*_1*i,*_ *y*_1*i*_) and (*x*_1*j*_, *y*_1*j*_) are coordinates of the primary coil vertex and (*x*_2*i*_, *y*_2*j*_) and (*x*_2*j*_, *y*_2*j*_) are the secondary coil vertex coordinates.

As indicated by Equation (20), the Neumann formula necessitates the specification of the vertex coordinates for each coil when calculating the mutual inductance between two arbitrarily positioned rectangular coils. However, if the positions of the coils change, the vertex coordinates must be recalculated, which increases the computational load and decreases computational efficiency.

**Figure 5:**
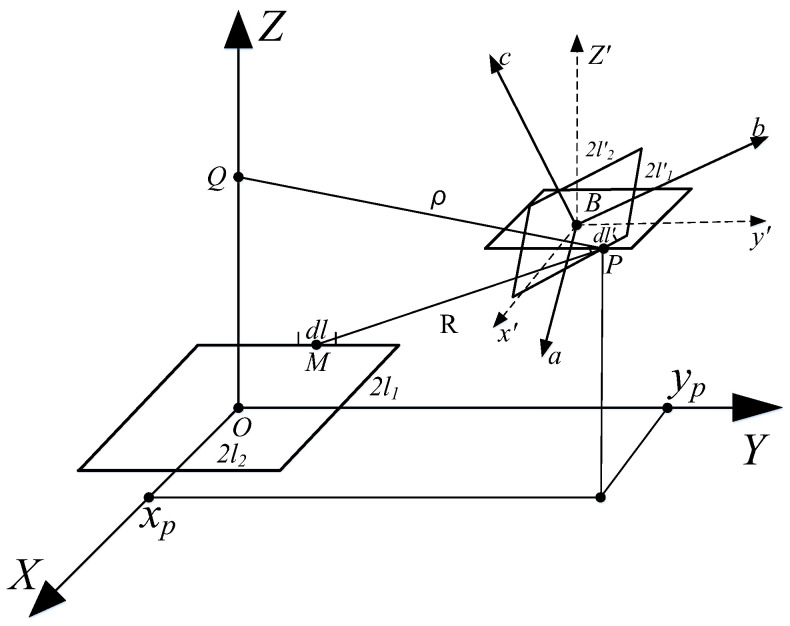
Schematic diagram of the Kalantarov–Zeitlin method.

As shown in Equation (21), the Neumann formula requires the vertex coordinates of each coil to be provided when calculating the mutual inductance between two rectangular coils positioned arbitrarily. However, if the positions of the coils change, the vertex coordinates must be recalculated, which increases computational load and reduces efficiency. As illustrated in Figure 5, let us select an arbitrary point *P* on the secondary coil, designated as *dl’*. Point P is connected to point *Q*, located on the *Z*-axis, by a line that is perpendicular to the *Z*-axis and has a length of *ρ*. We then create a circle with point *Q* as the center and *ρ* as the radius. This circle is parallel to the primary coil and is referred to as the *τ* circle. The segment dl’ is decomposed into *dz* along the *Z*-axis, *dρ* along the *ρ* direction, and dτ along the tangent direction of the *τ* circle. The expression for *dl’* is as follows:

(22) $$dl^{\prime }=dz+d\rho +d\tau$$

According to Equation (19), the mutual inductance between *dl’* and *dl* is as follows:

(23) $$dM=\frac{\mu_{0}}{4\pi }\frac{dl\cdot \left(d\tau +d\rho +dz\right)}{R}$$

**Figure 6:**
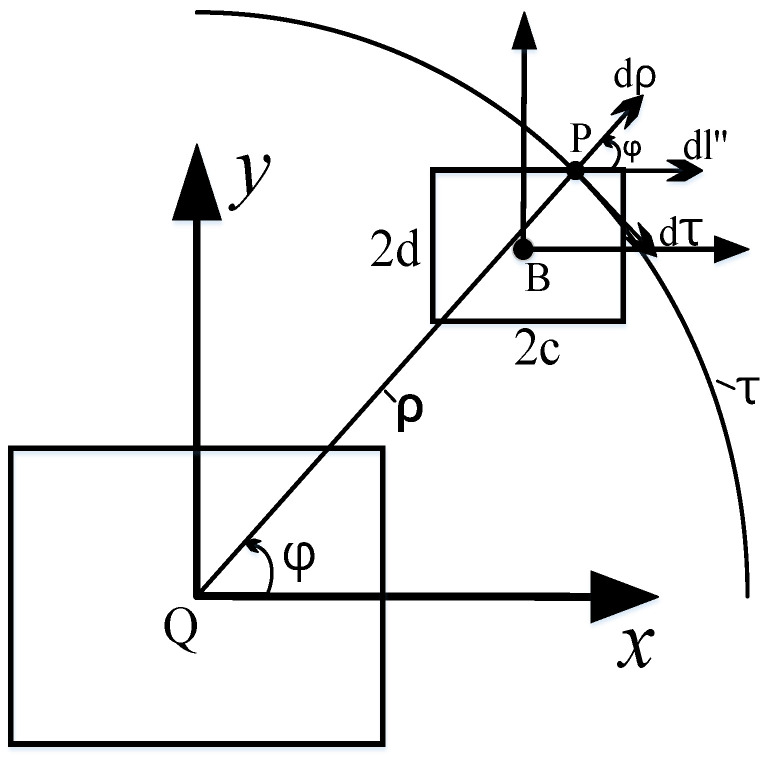
Relation diagram of *d***l″**, *d**ρ***, and *d**τ*** on the projection rectangle.

Based on the Kalantarov–Zeitlin method, the projection of the secondary coil onto the *τ*-circle plane is illustrated in Figure 6. Since the secondary coil is rectangular, its projection on the *τ*-circle plane appears as either a rectangle or a parallelogram, with the origin located at point *B*. The differential length, *dl″*, is expressed as follows:

(24) $$dl^{\prime \prime }=d\rho +d\tau$$

It can be seen from Equations (22) and (23) that:

(25) $$dM=\frac{\mu_{0}}{4\pi }\left(\frac{dl\cdot dl^{\prime \prime }+dl\cdot dz}{R}\right)$$

Since *d***z** is perpendicular to the plane of the primary coil, the mutual inductance *dM_z_* between *d***z** and the primary coil is zero; then the mutual inductance integral formula between the primary coil and the secondary coil is as follows:

(26) $$M=\frac{\mu_{0}}{4\pi }\oint_{l}\oint_{l^{\prime \prime }}\frac{dl\cdot dl^{\prime \prime }}{R}$$

where $R=\sqrt{{\left(x-x^{\prime \prime }\right)}^{2}+{\left(y-y^{\prime \prime }\right)}^{2}+{z^{\prime \prime }}^{2}}$. *D***l″** is the projection of d**l’** on the τ circular plane. Moreover, the x and y coordinates of the projection quadrilateral are identical to those of the secondary coil: *x’* = *x″*, *y’* = *y″*.

According to the coordinate transformation Equation (7), the *Z*-axis coordinate of the projected quadrilateral is as follows:

(27) $$z^{\prime \prime }=z_{B}-\sin\beta x^{\prime }+\left(\sin\alpha \cos\beta \right)y^{\prime }$$

Finally, the mutual inductance between two rectangular single-turn coils at arbitrary positions is obtained by the following formula:

(28) $$\begin{array}{l} M=\frac{\mu_{0}}{4\pi }\left[\int_{-l_{1}}^{l_{1}}dx\int_{-l_{1}^{\prime }cos\beta }^{l_{1}^{\prime }cos\beta }\frac{dx^{\prime \prime }}{\sqrt{{\left(x_{1}-x_{1}^{\prime \prime }\right)}^{2}+{\left(y_{1}-y_{1}^{\prime \prime }\right)}^{2}+{\left(z_{1}^{\prime \prime }\right)}^{2}}}+\int_{-l_{1}}^{l_{1}}dx\int_{l_{1}^{\prime }cos\beta }^{-l_{1}^{\prime }cos\beta }\frac{dx^{\prime \prime }}{\sqrt{{\left(x_{1}-x_{2}^{\prime \prime }\right)}^{2}+{\left(y_{1}-y_{2}^{\prime \prime }\right)}^{2}+{\left(z_{2}^{\prime \prime }\right)}^{2}}}\right.\\ \;+\int_{l_{1}}^{-l_{1}}dx\int_{-l_{1}^{\prime }cos\beta }^{l_{1}^{\prime }cos\beta }\frac{dx^{\prime \prime }}{\sqrt{{\left(x_{2}-x_{1}^{\prime \prime }\right)}^{2}+{\left(y_{2}-y_{1}^{\prime \prime }\right)}^{2}+{\left(z_{3}^{\prime \prime }\right)}^{2}}}+\int_{-l_{1}}^{l_{1}}dx\int_{l_{1}^{\prime }cos\beta }^{-l_{1}^{\prime }cos\beta }\frac{dx^{\prime \prime }}{\sqrt{{\left(x_{2}-x_{2}^{\prime \prime }\right)}^{2}+{\left(y_{2}-y_{2}^{\prime \prime }\right)}^{2}+{\left(z_{4}^{\prime \prime }\right)}^{2}}}\\ \;+\int_{-l_{2}}^{l_{2}}dy\int_{-l_{2}^{\prime }cos\alpha }^{l_{2}^{\prime }cos\alpha }\frac{dy^{\prime \prime }}{\sqrt{{\left(x_{3}-x_{3}^{\prime \prime }\right)}^{2}+{\left(y_{3}-y_{3}^{\prime \prime }\right)}^{2}+{\left(z_{5}^{\prime \prime }\right)}^{2}}}+\int_{-l_{2}}^{l_{2}}dy\int_{l_{2}^{\prime }cos\alpha }^{-l_{2}^{\prime }cos\alpha }\frac{dy^{\prime \prime }}{\sqrt{{\left(x_{3}-x_{4}^{\prime \prime }\right)}^{2}+{\left(y_{3}-y_{4}^{\prime \prime }\right)}^{2}+{\left(z_{6}^{\prime \prime }\right)}^{2}}}\\ \;\left.+\int_{l_{2}}^{-l_{2}}dy\int_{-l_{2}^{\prime }cos\alpha }^{l_{2}^{\prime }cos\alpha }\frac{dy^{\prime \prime }}{\sqrt{{\left(x_{4}-x_{3}^{\prime \prime }\right)}^{2}+{\left(y_{4}-y_{3}^{\prime \prime }\right)}^{2}+{\left(z_{7}^{\prime \prime }\right)}^{2}}}+\int_{l_{2}}^{-l_{2}}dy\int_{{l^{\prime }}_{2}cos\alpha }^{-{l^{\prime }}_{2}cos\alpha }\frac{dy^{\prime \prime }}{\sqrt{{\left(x_{4}-x_{4}^{\prime \prime }\right)}^{2}+{\left(y_{4}-y_{4}^{\prime \prime }\right)}^{2}+{\left(z_{8}^{\prime \prime }\right)}^{2}}}\right] \end{array}$$

Simplification of Equation (28) is as follows:

(29) $$M=\frac{\mu_{0}}{4\pi }\sum_{i=1}^{4}\sum_{j=1}^{4}{\left(-1\right)}^{i+j}\left[\varphi \left(x_{i},y_{i},z_{i};x_{j}^{\prime \prime },y_{j}^{\prime \prime },z^{\prime \prime }\right)+\varphi \left(y_{i},x_{i},z_{i};y_{j}^{\prime \prime },x_{j}^{\prime \prime },z^{\prime \prime }\right)\right]$$

(30) $$\begin{array}{ll} \varphi \left(x,y,z;x^{\prime \prime },y^{\prime \prime },z^{\prime \prime }\right)= & -\left(x-x^{\prime \prime }\right)\ln\left[\left(x-x^{\prime \prime }\right)+\sqrt{{\left(x-x^{\prime \prime }\right)}^{2}+{\left(y-y^{\prime \prime }\right)}^{2}+{\left(z^{\prime \prime }\right)}^{2}}\right]\\ & +\sqrt{{\left(x-x^{\prime \prime }\right)}^{2}+{\left(y-y^{\prime \prime }\right)}^{2}+{\left(z^{\prime \prime }\right)}^{2}} \end{array}$$

This calculation method allows for the projection of the secondary coil at any position within a specified plane, simplifying complex positional changes and reducing the calculation time for mutual inductance between coils. In the following sections, this article will compare the calculation time of this method with that of the Neumann formula and the magnetic flux density method.

From Equation (1), the electromagnetic force generated by the mutual inductance between the rectangular single-turn coils may not be sufficient to provide the force required for the unfolding of the solar sail panels. Therefore, it is essential to enhance the electromagnetic force by increasing the number of turns in the coils.

**Figure 7:**
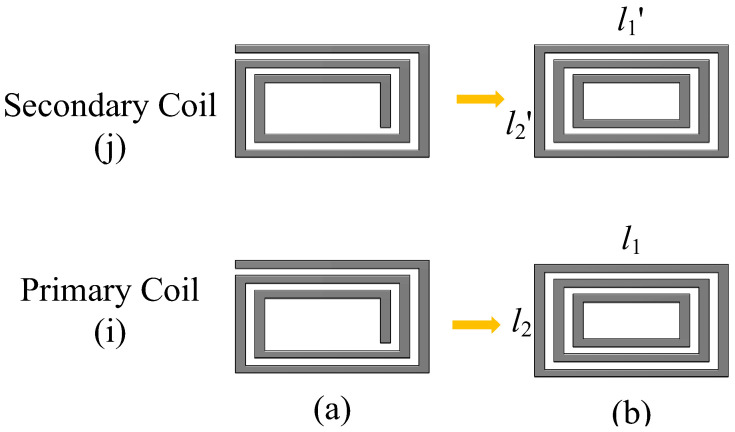
Simplification process of multi-turn spiral planar coils: (**a**) real spiral planar coils and (**b**) approximated form of the coils.

As illustrated in Figure 7, the mutual inductance of a multi-turn rectangular spiral coil is calculated by simplifying the planar spiral coil. The rectangular spiral coil (Figure 7a) is approximately modeled as several concentric single-turn rectangular coils (Figure 7b). Ultimately, the following formula for calculating the mutual inductance (*M_t_*) of the rectangular spiral coil is derived using the principle of superposition:

(31) $$M_{t}=\sum_{i=1}^{N_{1}}\sum_{j=1}^{N_{2}}M_{ij}$$

where *N*_1_ and *N*_2_ are the turns of the primary coil and the secondary coil, respectively. *M_ij_* is the mutual inductance calculation formula of single-turn rectangular coils (Equation (28)).

## 3. Algorithm Verification

In order to verify the accuracy of the calculation method for mutual inductance between rectangular coils positioned arbitrarily in space, the formula derived in this paper is compared with the magnetic flux density method and the Neumann formula, using calculation examples from published studies. In all calculations, the primary coil is located on the *XOY* plane, with its center at the origin *O* (0, 0, 0).

### 3.1. Calculation of Vertical Offset Mutual Inductance

**Figure 8:**
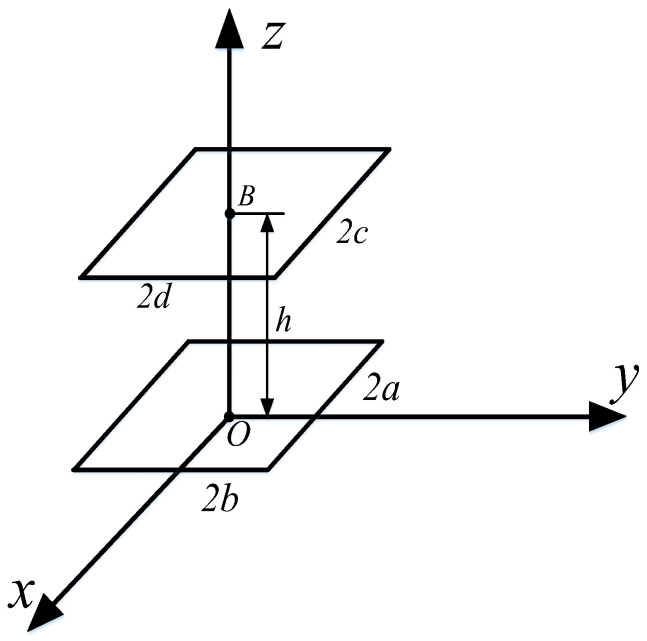
Schematic diagram of the position of two rectangular coils in the vertical direction.

As illustrated in Figure 8, the two rectangular coils are arranged in parallel and are coaxial, with a vertical separation of *h* between them. The length and width of the primary coil are designated as *l*_1_ = *a* and *l*_2_ = *b*, while the length and width of the secondary coil are represented as *l’*_1_ = *c* and *l’*_2_ = *d*. The origin of the secondary coil is defined at coordinates *x_B_* = *y_B_* = 0 and *z_B_* = *h*. The angles of rotation are set to *α* = *β* = *γ* = 0.

(1) Example 1

E. R. Joy et al. [32] calculated the mutual inductance between multi-turn rectangular coils using the magnetic flux density method. The dimensions of the two quadrilateral coils are 18 cm by 18 cm. The number of turns for the primary coil (*N*_1_) is 11, while the number of turns for the secondary coil (*N*_2_) is 9. The vertical distance (*h*) is increased from 2 cm to 10 cm in increments of 1 cm. The results of the calculations are presented in Table 2.

According to Table 2, the calculations obtained using the magnetic flux density method are slightly lower than the results presented in this paper, with an average relative error of 0.83%. In contrast, the Neumann formula is more accurate and nearly identical to the method proposed in this paper, exhibiting an average relative error of 0.46%.

### 3.2. Calculation of Horizontal Offset Mutual Inductance

**Figure 9:**
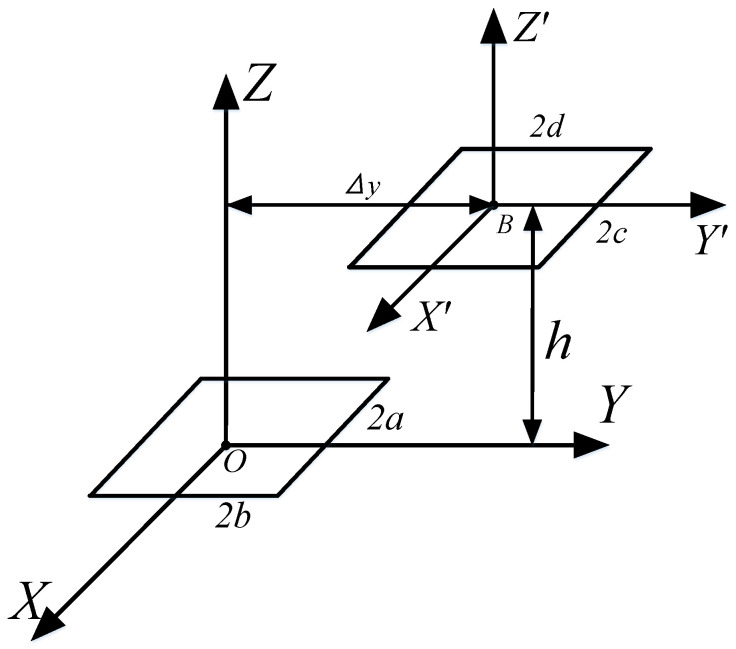
Schematic diagram of two rectangular coils in a horizontal direction.

In Figure 9, the secondary coil shifts horizontally, while the vertical distance *h* between the two coils remains constant. The variable *Δy* represents the distance the secondary coil moves along the *Y*-axis.

(1) Example 2

Taking reference [32] as an example, the side length of the square coil is 2*a* = 18 cm, with the number of turns for coil *N*_1_ = 11 and *N*_2_ = 9. The vertical height between the coils is *h* = 2.1 cm, and *Δy* increases from 1 cm to 100 cm in increments of 1 cm. The results are presented in the table below. It can be observed from Table 3 that under the conditions of this model.

The computational error is smaller when moving horizontally than when moving vertically. The average relative error of the method proposed in this paper is 0.56% when compared directly to the magnetic flux density method and only 0.21% when compared to the Neumann formulation.

(2) Example 3

Li [33] calculated the mutual inductance between single-turn rectangular coils using the magnetic flux density method and extended his findings to multiple-turn rectangular coils through the principle of superposition. The dimensions of both the primary and secondary coils are identical: *2a* = *2c* = 120 mm and *2b* = *2d* = 100 mm. The number of turns is *N*_1_ = 12 for the primary coil and *N*_2_ = 10 for the secondary coil, with a vertical separation of *h* = 80 mm. The variable *Δy* increases from 10 mm to 100 mm in increments of 10 mm, and the results of these calculations are presented in Table 4. Under these conditions, the maximum error between the method proposed in this paper, the magnetic flux density method, and the Neumann formula is less than 2%.

### 3.3. Mutual Inductance Calculation at Arbitrary Position in Space

The variation in mutual inductance between the coils during angular rotation is particularly critical for the deployment of solar sail panels. This section compares the mutual inductance between the two coils during angular rotation at various positions, using the method proposed in this paper and case studies from the published literature. The angles α, *β*, and *γ* represent the rotation angles of the secondary coil, ranging from 0 to 360°.

(1) Example 4

Taking reference [33] as an example, the coil parameters are identical to those in Example 4. The center of the secondary coil is positioned at *x_B_* = *y_B_* = 0, *z_B_* = 80 mm, and the secondary coil is deflected around the X’-axis, forming an included angle α with the plane of the primary coil. The calculated deflection angle *α*, as the mutual inductance increases from 0° to 90°, is presented in the table below.

From Table 5, the calculation method presented in this paper is largely consistent with the magnetic flux density method and the Neumann formula when the secondary coil is in rotation. The relative error between the magnetic flux density method and the approach proposed in this paper is 0.63%, while the relative error between this method and the Neumann formula is 1.03%. These results indicate that the calculation method proposed in this paper can accurately determine the mutual inductance of the coil during rotation.

(2) Example 5

According to reference [26], the side lengths of the primary coil and the secondary coil are 2*a* = 28 cm and 2*c* = 17 cm, respectively. The coordinates of the origin B of the secondary coil are *x_B_* = 0, *y_B_* = 4.33 cm, and *z_B_* = 17.5 cm. The secondary coil is rotated at an arbitrary angle in this position, and the calculated mutual inductance is presented in Table 6. This table indicates that when the center of the secondary coil is positioned arbitrarily in space, the calculation formula derived in this paper is largely consistent with the results obtained from the Neumann formula, exhibiting a mean square error of 0.074%.

The calculation formula developed in this study closely aligns with the values obtained using the magnetic flux density method and the Neumann formula, demonstrating an error margin of less than 1%, suggesting the reliability of the calculation formula.

**Figure 10:**
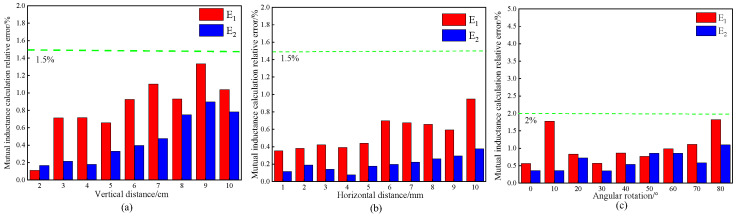
Calculation error of mutual inductance under various operating conditions: (**a**) vertical direction; (**b**) horizontal direction; and (**c**) angular rotation.

In order to verify the accuracy of the mutual inductance calculation method proposed in this paper, the relative error between the method of this paper and the magnetic flux density method is denoted as *E*_1_, and the relative error with the Neumann formula is denoted as *E*_2_. Figure 10a illustrates the relative error plot for vertical movement. It is evident that when the secondary coil varies from 2 to 10 cm, the error in all mutual inductance calculations remains below 2%, with the error using the Neumann formula even falling below 1%. Figure 10b presents the relative error plot of mutual inductance during horizontal movement, indicating that the calculation error in the horizontal direction is slightly smaller than that in the vertical direction, with the relative error remaining below 1.5%. Figure 10c depicts the calculated relative error in mutual inductance between the primary and secondary coils during a rotation of 0 to 90 degrees. It is observed that the error for angular rotation is marginally larger compared to the horizontal and vertical directions; however, the relative error still remains within 2%. This demonstrates that the calculation method proposed in this paper is reliable.

**Figure 11:**
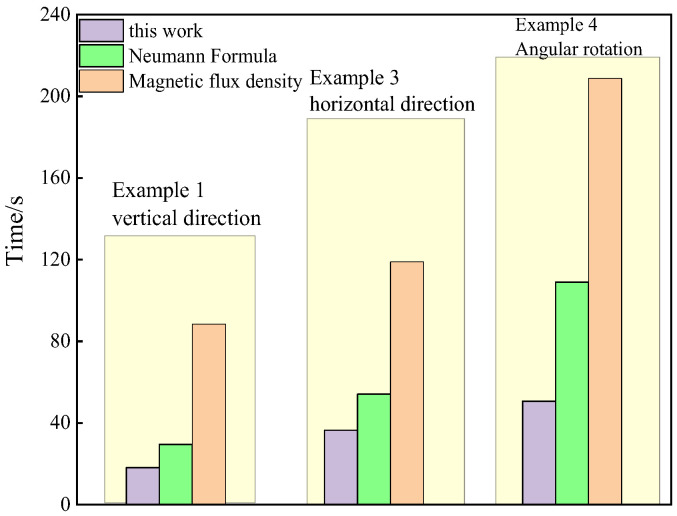
Comparison diagram of mutual inductance calculation time.

To illustrate the simplicity of the formula derived in this paper, the tic and toc functions in MATLAB2019 are utilized to obtain the code execution time, as shown in Figure 11. This figure demonstrates that the mutual inductance calculation time for the formula presented in this paper is the shortest under three conditions: horizontal, vertical, and rotational angles. In the horizontal and vertical orientations, the calculation times for the proposed method are comparable to those of the Neumann formula, with time differences of 4.3 s and 4.7 s, respectively. However, when the secondary coil is rotated, the calculation time for this method is significantly reduced to 50.7034 s, which is considerably lower than that of the Neumann formula (109.0374 s) and the magnetic flux density method (208.7593 s). This result underscores the efficiency of the formula derived in this paper.

## 4. Experimentation and Numerical Simulation

In this section, we aim to further validate the accuracy of the mutual inductance calculation formula for multi-turn rectangular coils in the context of electromagnetic force acquisition. To achieve this, we conduct experimental measurements and numerical simulations utilizing a rectangular helical coil.

### 4.1. Mutual Inductance Measurement Experiment

**Figure 12:**
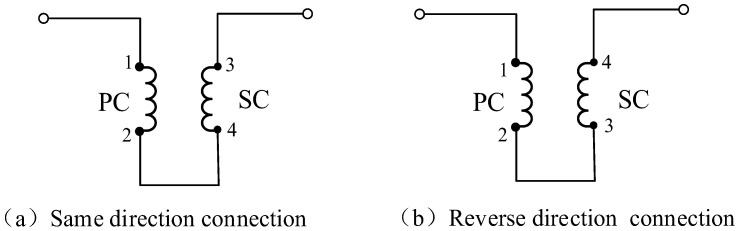
Schematic diagram of mutual inductance measurement experiment: (**a**) same direction connection and (**b**) reverse direction connection.

The experimental measurement principle of mutual inductance differs from the theoretical calculation method. As illustrated in Figure 12, mutual inductance is determined by calculating the inductance difference between the primary coil (PC) and the secondary coil (SC) when they are connected in series, both in the same and in reverse directions. *L*_T_ and *L*_R_ represent the self-inductance of the PC and SC, respectively. In Figure 12a, the coils are connected in the same direction, resulting in an inductance of *L*_1_ = *L_T_* + *L_R_* + 2*M* at both ends of the line. In Figure 12b, the coils are connected in the reverse direction, yielding an inductance of *L*_2_ = *L_T_* + *L_R_* − 2*M* at both ends of the line. Therefore, the mutual inductance between the PC and SC can be expressed as *M* = |*L*_1_ − *L*_2_|/4.

**Figure 13:**
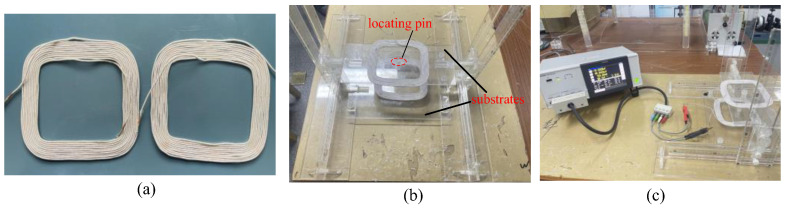
Mutual inductance experiment diagram: (**a**) rectangular spiral coil; (**b**) acrylic mobile platform; and (**c**) impedance analyzer.

The rectangular spiral coil is illustrated in Figure 13a, where each coil is equipped with two connectors for wiring connections. The main parameters of the rectangular spiral coil are detailed in Table 7. To obtain experimental values for the mutual inductance between the rectangular coils at various positions, a mutual inductance measurement platform has been constructed, as shown in Figure 13b,c. This experimental platform primarily consists of an IM3536 impedance analyzer and a custom-made acrylic base. The impedance analyzer is used to measure the self-inductance of the coil, while the acrylic base allows for adjustments in the position of the secondary coil. During the experiment, both the primary and secondary coils are secured at the center of the substrate using locating pins, ensuring that the center of the secondary coil remains fixed during positional adjustments.

To minimize electromagnetic interference from external factors while measuring mutual inductance during the experiment, the entire setup was conducted on a non-magnetic wooden table and housed within an acrylic frame. This approach aimed to mitigate the influence of the surrounding environment on the measurement results. Additionally, the long terminals connected to the impedance analyzer were twisted during the experiment to reduce the impact of the current flowing through the terminals on the electromagnetic field. The measurement results were further refined by calculating the average value from multiple measurements, resulting in more accurate outcomes.

**Figure 14:**
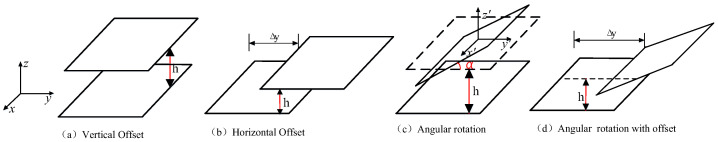
Schematic diagram of coil position change: (**a**) vertical direction; (**b**) horizontal direction; (**c**) angular rotation; and (**d**) angular rotation with offset.

Considering the attitude change in the solar sail panel during deployment, four mutual sensing measurement attitudes are designed as shown in Figure 14.

Here, *h* is the distance between the primary coil and the secondary coil in the vertical direction, *α* is the angular rotation of the secondary coil, and *Δy* is the horizontal offset of the secondary coil along the Y’-axis.

### 4.2. Numerical Simulation of Mutual Inductance

**Figure 15:**
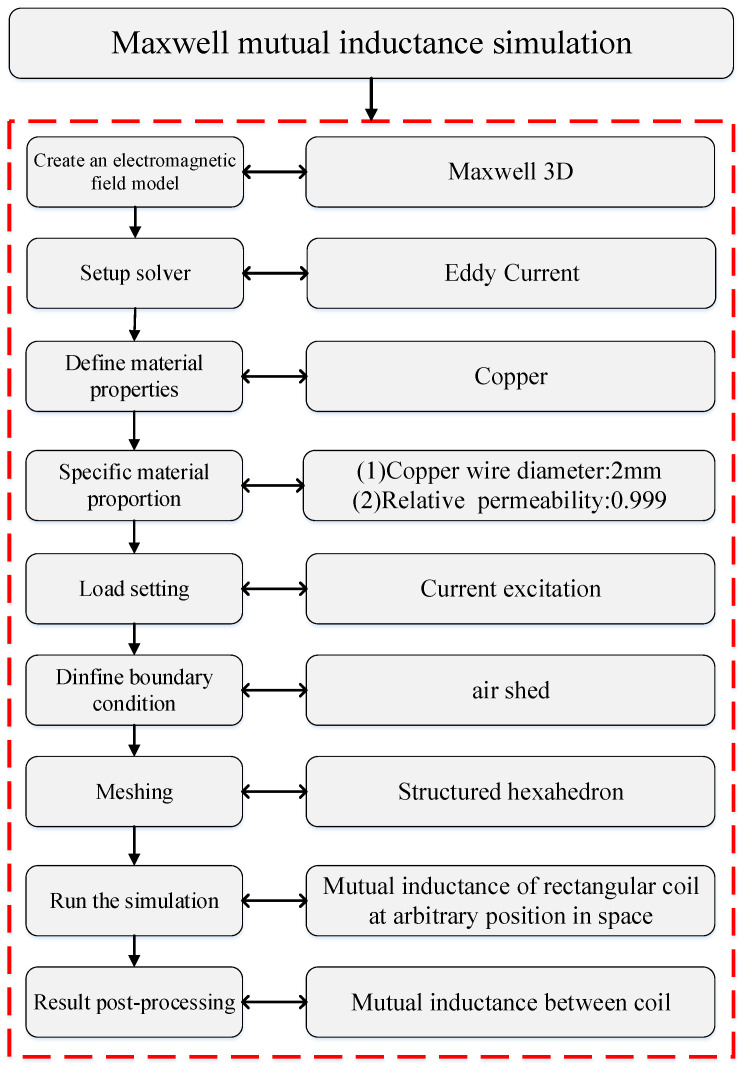
Mutual inductance simulation flow chart.

In order to verify the accuracy of the mutual inductance calculations presented in Equations (28) and (31), this paper utilizes ANSYS Electronics2021 for simulation validation. Based on the experimental parameters, a mutual inductance simulation model of the rectangular coil has been established, as illustrated in Figure 15.

The finite element model consists of a primary and a secondary rectangular helical coil. The model is created using SolidWorks2013 and imported into ANSYS Maxwell. The model parameters are consistent with those used in mutual inductance experiments. During the analysis phase, eddy currents are considered, and the coil is divided along the YZ plane to isolate the current-carrying section, to which current excitation is applied. In the boundary condition settings, a vacuum region is established to simulate the space environment, extending from 300 to 500 times the size of the model. In the numerical simulation, the position of the primary coil remains constant, while the relative position between the coils is modified by adjusting the position and orientation of the secondary coil.

## 5. Results and Discussion

### 5.1. Comparison of Calculation and Experimental Results

(1) Influence of vertical offset on mutual inductance

In Figure 14a, the secondary coil is moved along the positive *Z*-axis in increments of 10 mm while keeping the position of the primary coil fixed. The vertical distance *h* between the coils increases from 10 mm to 100 mm, and the mutual inductance of the coils is shown in Table 8.

*M*_c_ represents the calculated value of mutual inductance derived from the mutual inductance calculation formula presented in this paper, while *M*_e_ denotes the experimentally measured value. The experimental error, *ε*_e_, is expressed as follows:

(32) $$\varepsilon_{e}=\frac{\left|M_{c}-M_{e}\right|}{M_{e}}\times 100\%$$

It can be observed from the table that when the vertical distance *h* ranges from 10 mm to 100 mm, the calculated values of mutual inductance closely correspond to the experimental values, exhibiting an experimental error of approximately 2%. As the distance increases, the mutual inductance of the coil gradually decreases, and the calculated results align well with the actual observations.

(2) Influence of horizontal offset on mutual inductance

The vertical offset remains unchanged, preserving the position of the primary coil. As illustrated in Figure 14b, the vertical distance between the coils remains constant at *h* = 80 mm. The secondary coil shifts horizontally in increments of 10 mm, moving from 50 mm in the positive direction of the *Y*-axis to −50 mm. The mutual inductance between the coils is presented in Table 9.

The experimental error is approximately 2% when the horizontal offset varies from 50 mm to −50 mm. The calculated results align closely with the experimental findings, indicating that Equation (31) can accurately determine the mutual inductance between coils as they move horizontally.

(3) Influence of angular rotation on mutual inductance

The vertical distance *h* between the two coils remains constant at 80 mm. Figure 14c illustrates the schematic diagram of the secondary coil rotating around the X’-axis. The angle *α* increases from 0° to 90°, and the mutual inductance of the coil is presented in Table 10. It is observed that the experimental error remains below 2% during the transition of the angle from 0° to 90°, and the calculations align with the experimental results. When *α* < 45°, the mutual inductance remains constant as the angle increases. However, when *α* > 45°, the magnetic linkage through the secondary coil gradually decreases with the increase in *α*, resulting in a decline in mutual inductance.

(4) Influence of angular rotation with offset on mutual inductance

As illustrated in Figure 14d, the position and angle of the secondary coil are adjusted using a custom-made acrylic stand. The secondary coil is moved 40 mm in the positive direction along the *Y*-axis while maintaining a distance of 80 mm between the two lines. Table 11 presents the calculated and experimental values of mutual inductance between rectangular coils as the angle *α* is increased from 0° to 90°.

It can be observed from the table that the experimental error (*ε*_e_) is less than 2% when the rotation angle varies from 0° to 90°. Additionally, the calculated results are generally consistent with the experimental findings.

### 5.2. Comparison of Calculation with Experimental and Simulation Results

**Figure 16:**
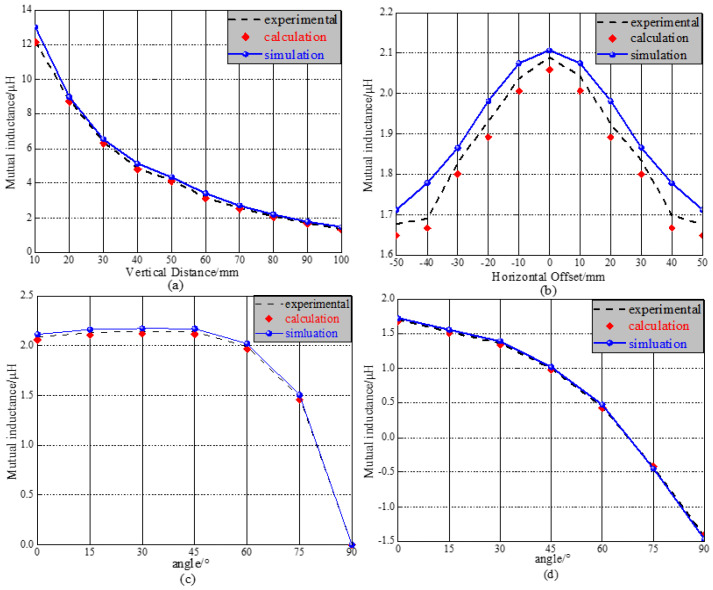
Comparison diagram of mutual inductance calculation, experiment, and simulation: (**a**) vertical offset; (**b**) horizontal offset; (**c**) angular rotation; and (**d**) angular rotation with offset.

Figure 16a illustrates the variation of mutual inductance in relation to vertical offset. This figure shows that mutual inductance gradually decreases as the vertical distance increases. This decline occurs because the magnetic coupling between the coils weakens with greater vertical separation, leading to a reduction in mutual inductance. When the vertical distance h changes from 10 mm to 100 mm, the average error between the calculated and simulated results is 5.8%, while the error between the calculated and experimental results is 1.5%. This indicates that Equation (31) accurately calculates mutual inductance in the vertical direction. Figure 16b illustrates the variation in mutual inductance as a result of a horizontal offset. Initially, the mutual inductance between the coils increases, followed by a decrease, exhibiting a symmetrical trend. This behavior is attributed to the symmetrical overlap areas between the coils, which lead to a corresponding symmetrical trend in their coupling. As the horizontal offset (*Δy*) varies from −50 mm to 50 mm, the average errors between the simulation and experimental results are 4.1% and 1.7%, respectively. The calculations, along with the experimental and simulation results of mutual inductance, demonstrate excellent consistency.

The relationship between angle rotation and coil mutual inductance is illustrated in Figure 16c. The vertical axis represents the secondary coil rotating around the X’-axis in increments of 15°. The curve in this figure shows that when the angle *α* is less than 45°, the mutual inductance between the coils changes only slightly. However, when *α* exceeds 45°, the magnetic linkage decreases as *α* increases, leading to a reduction in mutual inductance. At *α* equal to 90°, the mutual inductance is nearly reduced to zero. This occurs because, when the two coils are perpendicular to each other, the magnetic linkage passing through the secondary coil approaches zero, resulting in a significant decline in the magnetic induction intensity of the coil and ultimately causing the mutual inductance to become zero. The calculated results for mutual inductance align closely with both experimental and simulation findings. Figure 16d illustrates the variation curve of mutual inductance as a function of coil angle rotation with positional offset. Unlike angle rotation, the horizontal offset between the coils results in the absence of a plateau period for mutual inductance when *α* is less than 45°. Instead, the mutual inductance value gradually decreases as the angle increases, indicating that the positional offset has a more significant impact on mutual inductance than angular rotation. When the angle between the two coils reaches 75°, the mutual inductance becomes negative. Throughout the angle change process, the average error of the simulation is approximately 5.3%, while the average error of the experiment is 2.1%. The simulation results and calculations are largely consistent.

**Figure 17:**
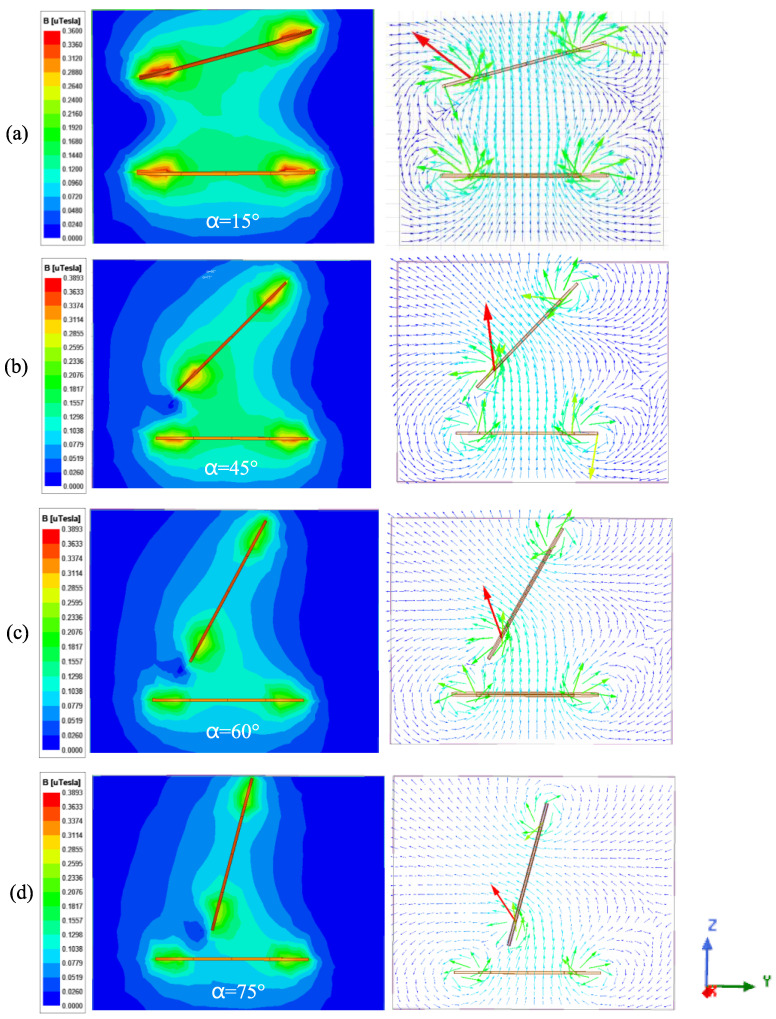
Magnetic induction intensity nephogram between coils at angular rotation: (**a**) No. 23; (**b**) No. 25; (**c**) No. 26; and (**d**) No. 27.

It can be observed from the magnetic induction intensity and the magnetic field distribution cloud diagram during the rotation of the angle (Figure 17) that when *α* is at 15° and 45°, the magnetic field strength is approximately 0.35 μT. The change in coupling magnetic linkage between the coils is minimal, and the mutual inductance between the coils remains essentially unchanged. However, when the angle increases to 60°, the magnetic induction intensity decreases to 0.18 μT. The arrow in the figure indicates the direction of the magnetic induction line of the coil.

**Figure 18:**
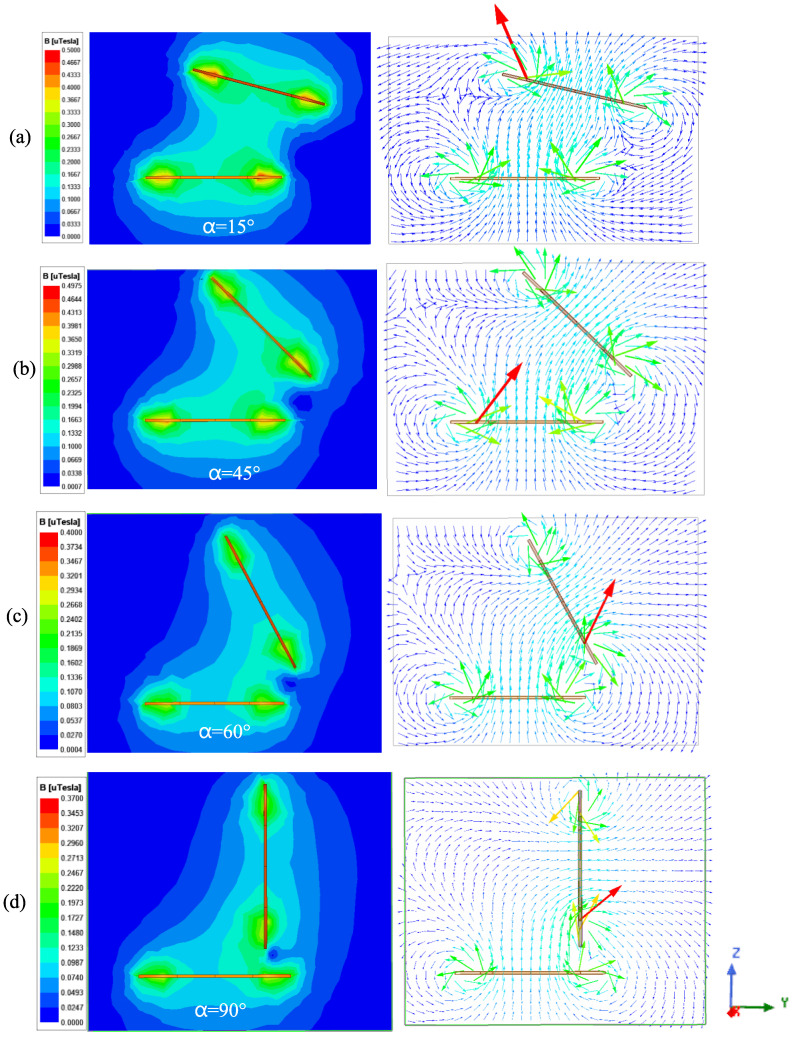
Magnetic induction intensity nephogram between coils during angular deflection and offset: (**a**) No. 30; (**b**) No. 32; (**c**) No. 33; and (**d**) No. 35.

Figure 18 illustrates the intensity of magnetic induction and the distribution of the magnetic field following the angular rotation of the coil with an offset. Unlike angular rotation, the horizontal offset between the coils causes the magnetic linkage to gradually decrease as the angle *α* increases from 0° to 90°. This results in a corresponding decline in mutual inductance. According to the magnetic field distribution diagram, when *α* reaches 90°, some magnetic linkage between the coils remains; however, the direction of this magnetic linkage has reversed, resulting in negative mutual inductance between the coils.

### 5.3. Mutual Inductance Verification Between Coils with Different Turns

In the previous section, the accuracy of the theoretical calculation of mutual inductance between coils with an equal number of turns was verified. To further evaluate the applicability of the derived formula, this section simulates and validates the theoretical calculation of mutual inductance between coils with varying numbers of turns. Based on the coil parameters used in the experiment, the number of turns in the secondary coil is adjusted to 5, 15, and 20 turns. The mutual inductance between the coils is then simulated using ANSYS Maxwell and Equation (31).

**Figure 19:**
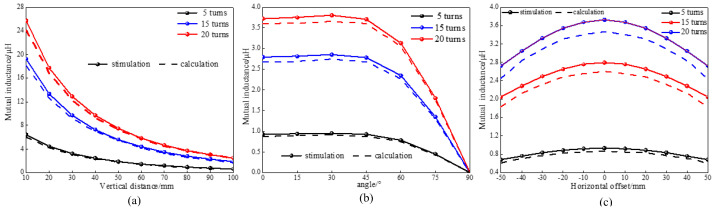
Comparison diagram of mutual inductance between coils with different turns: (**a**) vertical distance; (**b**) horizontal distance; and (**c**) angular rotation.

Figure 19a illustrates the mutual inductance curve between the secondary coil and the primary coil as the number of turns varies in the vertical direction. The horizontal axis represents the vertical distance in 10 mm intervals, while the vertical axis indicates mutual inductance, measured in microhenries (μH). The theoretical values of mutual inductance depicted in this figure closely align with the simulation results. The maximum error occurs at a distance of 10 mm, with an approximate error of 6%. As the vertical distance increases, the discrepancy between the theoretical calculations and the simulation results gradually diminishes, with the two nearly coinciding at a distance of 100 mm. Figure 19b presents the mutual inductance curve between the secondary coil and the primary coil as the secondary coil rotates around the *x*-axis for different numbers of turns, with a rotation angle interval of 15°. This figure shows that when the rotation angle of the secondary coil is less than 60°, the mutual inductance between the coils remains close to its maximum value, and the theoretical calculations and simulation results exhibit strong consistency. As the angle increases, the gap between theoretical and simulation values gradually narrows. Figure 19c depicts the mutual inductance curve between the secondary coil and the primary coil with varying turns during horizontal movement. The theoretical values of mutual inductance are in good agreement with the experimental measurements. Similarly, as the horizontal distance increases, the difference between the theoretical calculations and the simulation results decreases. From the simulation results presented in the three figures above, it is evident that the calculation error is approximately 6%. Therefore, the mutual inductance calculation formula for rectangular coils, as derived in this paper, is applicable to the mutual inductance between coils with varying numbers of turns.

Through experiments and simulations, the calculation method proposed in this paper accurately determines the mutual inductance between two rectangular coils with varying numbers of turns and arbitrary positions. This method ensures the effective configuration of the space electromagnetic drive structure and facilitates the control of electromagnetic force during the unfolding process of windsurfing.

## 6. Conclusions

In order to accurately capture the electromagnetic force and provide for the electromagnetic force to drive the space solar sail panels. A fast and precise method for calculating the mutual inductance between rectangular coils at arbitrary spatial locations is essential. In this paper, we propose a theoretical calculation method for the mutual inductance between two rectangular coils positioned at any orientation in space, based on the Neumann formula and the Kalantarov–Zeitlin method, utilizing the spatial rectangular coordinate transformation formula. Building on this foundation, we derive a calculation method for the mutual inductance of rectangular multi-turn coils by applying the principle of superposition. Compared with the existing mutual inductance calculation methods, the method proposed in this paper utilizes the Kalantarov–Zeitlin method to project the coils, which can obtain the position of the rectangular coils in the spatial coordinates more quickly, simplify the calculation steps of the coordinates of the vertices of the coils in the space, and speed up the mutual inductance calculation.

The accuracy and computational efficiency of the proposed method are compared with those of other methods through calculation examples from the published literature. The results indicate that the relative error between the proposed method and the alternatives remains within 2%, while the computation time is significantly shorter than that of the competing methods. To further validate the accuracy of the calculation method presented in this paper, we analyze the attitude changes in a solar sail panel during deployment, including horizontal offset, vertical offset, angular offset, and angular rotation with offset. Mutual inductance measurements and simulations are conducted using two rectangular multi-turn coils. The findings demonstrate that the calculated values of mutual inductance from the proposed method closely align with the simulated and experimental values, exhibiting an error of approximately 1.5%. This strongly supports the accuracy of the mutual inductance calculation formula derived in this paper. Additionally, the method proves beneficial for vibration control of solar panel structures and for acquiring electromagnetic force in electromagnetic docking applications. At the same time, there may be strong electromagnetic interference in space, so the accuracy and simplicity of the mutual inductance calculation method in this paper under electromagnetic interference need to be improved in the subsequent work.

## Figures and Tables

**Table 1:** Nomenclature.

Symbols	Meanings	Symbols	Meanings
*α*, *β*, *γ*	Euler angles	*M_t_*	Mutual inductance of a multi-turn coil
*R_αβγ_*	Coordinate transformation matrix	*dρ*, *dτ*	Radius and tangent of projected circular plane
*B*	Magnetic induction intensity	*dl*, *l*_1_, *l*_2_	Primary coil side length
*A*	Vector magnetic potential	*dl’*, *l’*_1_, *l’*_2_	Side length of secondary coil
*ψ*	Magnetic linkage	*a*, *b*	Side length of primary coil of example model
*M*	Mutual inductance	*c*, *d*	Side length of secondary coil of example model
*N*_1_	Turns of the primary coil	*h*	Vertical height
*N*_2_	Turns of the secondary coil	*Δy*	Horizontal displacement

**Table 2:** Calculation of mutual inductance for example 1.

Vertical Distance *h*/cm	*M*/μH (Magnetic Flux Density)	*M*/μH (Neumann Formula)	*M*/Μh (This Work’s Equation (31))
2	18.05	18.08	18.10
3	13.97	14.00	14.10
4	11.16	11.18	11.26
5	9.11	9.14	9.20
6	7.54	7.57	7.64
7	6.32	6.35	6.42
8	5.34	5.38	5.43
9	4.46	4.50	4.56
10	3.83	3.86	3.90

**Table 3:** Calculation of mutual inductance for example 2.

Horizontal Distance *Δy*/mm	*M*/μH (Magnetic Flux Density)	*M*/μH (Neumann Formula)	*M*/μH (This Work’s Equation (31))
1	17.04	17.08	17.10
2	15.76	15.79	15.82
3	14.24	14.28	14.30
4	12.75	12.79	12.80
5	11.35	11.38	11.40
6	10.04	10.09	10.11
7	8.88	8.92	8.94
8	7.61	7.64	7.66
9	6.75	6.77	6.79
10	5.27	5.30	5.32

**Table 4:** Calculation of mutual inductance for example 3.

Horizontal Distance *Δy*/mm	*M*/μH (Magnetic Flux Density)	*M*/μH (Neumann Formula)	*M*/μH (This Work’s Equation (31))
10	2.7281	2.7369	2.7587
20	2.6498	2.6793	2.6891
30	2.5244	2.5413	2.5510
40	2.3586	2.3719	2.3767
50	2.1606	2.1724	2.1883
60	1.9395	1.9577	1.9675
70	1.7045	1.7233	1.7531
80	1.4646	1.4769	1.4868
90	1.2279	1.2413	1.2482

**Table 5:** Calculation of mutual inductance for example 4.

Deflection Angle *α*/°	*M*/μH (Magnetic Flux Density)	*M*/μH (Neumann Formula)	*M*/μH (This Work’s Equation (31))
0	2.7535	2.7591	2.7691
10	2.7301	2.7687	2.7787
20	2.7705	2.7735	2.7935
30	2.8181	2.8241	2.8341
40	2.8141	2.8232	2.8385
50	2.6765	2.6741	2.6971
60	2.323	2.3259	2.3459
70	1.7119	1.721	1.731
80	0.8948	0.9012	0.9111
90	0	0	0

**Table 6:** Calculation of mutual inductance for example 5.

α/rad	β/rad	γ/rad	*M*/μH (Neumann Formula)	*M*/μH (This Work’s Equation (28))
π/3	0	π/6	12.66	12.476
π/4	π/3	π/3	14.64	14.519
π/3	π/4	π/2	15.70	15.505
π/4	π/5	2π/3	23.92	23.417
π/5	π/6	5π/6	26.16	25.935

**Table 7:** Main parameters of the coil.

Coil Parameters	Size	Material Quality
Length of the innermost circle/mm	100	copper wire
Innermost ring width/mm	80	
Turns of the primary coil	10	
Turns of the secondary coil	10	
Diameter/mm	2	

**Table 8:** Mutual inductance for vertical misalignment.

No.	Vertical Distance *h*/mm	*M*_e_/μh	*M*_c_/μh	*ε*_e_/%
1	10	12.1632	12.0112	1.25
2	20	8.7757	8.6539	1.39
3	30	6.3631	6.2723	1.43
4	40	4.8511	4.7783	1.50
5	50	4.1551	4.100	1.33
6	60	3.1863	3.126	1.89
7	70	2.5664	2.5193	2.15
8	80	2.0895	2.0591	1.46
9	90	1.7034	1.6718	1.86
10	100	1.3559	1.3355	1.51

**Table 9:** Mutual inductance for horizontal offset.

No.	Horizontal Offset *Δy*/mm	*M*_e_/μh	*M*_c_/μh	*ε*_e_/%
11	−50	1.6777	1.6490	1.71
12	−40	1.6905	1.6677	1.35
13	−30	1.8300	1.8008	1.6
14	−20	1.9309	1.8916	2.04
15	−10	2.0366	2.0067	1.47
16	0	2.0895	2.0591	1.45
17	10	2.0426	2.0067	1.77
18	20	1.9243	1.8916	1.76
19	30	1.8346	1.8008	1.84
20	40	1.6993	1.6677	1.86
21	50	1.6993	1.6490	1.58

**Table 10:** Mutual inductance for angular rotation.

No.	α/°	*M*_e_/μh	*M*_c_/μh	*ε*_e_/%
22	0	2.0895	2.0591	1.46
23	15	2.1293	2.1079	1.01
24	30	2.1465	2.1196	1.25
25	45	2.1400	2.1142	1.20
26	60	1.9903	1.965	1.27
27	75	1.4824	1.4596	1.54
28	90	0.00095	0	/

**Table 11:** Mutual inductance for vertical rotation with horizontal offset.

No.	Secondary Coil Center Coordinates (0,40,80)/mm			
	*α*/°	*M*_e_/μh	*M*_c_/μh	*ε*_e_/%
29	0	1.6993	1.6677	1.86
30	15	1.5223	1.5015	1.37
31	30	1.3564	1.3301	1.2
32	45	0.9949	0.9719	1.31
33	60	0.44	0.4234	1.5
34	75	−0.4225	−0.4169	1.33
35	90	−1.4244	−1.3991	1.77

## Data Availability

Data is contained within the article.
